# Low-dose nicardipine during cardiopulmonary bypass improves carotid hemodynamics in CABG patients: a randomized controlled trial

**DOI:** 10.1186/s13019-025-03733-y

**Published:** 2025-12-24

**Authors:** Duanqi Zhu, Ying Han, Xiao Zhou, Zhenghong Wang, Bei Sun, Hongwei Shi, Yamei Zhao

**Affiliations:** 1 Department of Anesthesiology, Perioperative and Pain Medicine, Nanjing First Hospital, Nanjing Medical University, Nanjing, Jiangsu China; 2 Department of Anesthesiology, Children’s Hospital of Jiangnan University (Wuxi Children’s Hospital), Wuxi, Jiangsu China

**Keywords:** Internal carotid blood flow, Regional cerebral oxygen saturation

## Abstract

**Background:**

Postoperative cognitive dysfunction (POCD) is a common complication of coronary artery bypass grafting (CABG) with cardiopulmonary bypass (CPB), largely associated with cerebral hemodynamic disturbances. Carotid duplex ultrasonography–derived parameters, including peak systolic velocity (PSV-ICA) and end-diastolic velocity (EDV-ICA), provide reliable, noninvasive indicators of cerebral blood flow. This study evaluated whether intraoperative nicardipine infusion improves carotid blood flow and mitigates postoperative cognitive decline.

**Methods:**

In this randomized, double-blind trial, 64 elderly patients undergoing CABG were assigned to receive either continuous low-dose nicardipine (0.2–0.5 µg/kg·min) during CPB (nicardipine group, *n* = 32) or saline (control group, *n* = 32). The co-primary endpoints were intraoperative PSV-ICA and EDV-ICA at four predefined time points (T1: pre-anesthesia; T2: surgery initiation; T3: during CPB; T4: post-CPB). Secondary endpoints included regional cerebral oxygen saturation (rScO₂), plasma neuron-specific enolase (NSE), Mini-Mental State Examination (MMSE) scores, vasopressor use, ventilation time, ICU stay, and hospitalization length.

**Results:**

Baseline and intraoperative characteristics were comparable. Both groups showed reduced carotid flow velocities during CPB, but recovery of PSV-ICA at the end of CPB was significantly greater with nicardipine (63.7 ± 15.2 vs. 54.3 ± 13.9 cm/s; *P* = 0.025). EDV-ICA exhibited a similar trend without significant between-group differences. Secondary outcomes (MMSE, NSE) did not differ, and no adverse events occurred.

**Conclusion:**

Continuous low-dose nicardipine during CPB significantly improved the recovery of PSV-ICA without affecting systemic hemodynamics or increasing adverse events. These findings suggest a potential intraoperative neuroprotective role of nicardipine, warranting confirmation in larger trials with long-term cognitive outcomes.

## Introduction

Neurological and cognitive impairments are common complications after cardiac surgery and are associated with reduced quality of life and poor prognosis [1]. Although coronary artery bypass grafting (CABG) improves survival, it is frequently complicated by postoperative delirium and cognitive decline [2].

Adequate cerebral perfusion is essential for maintaining cognitive function [3]. Cardiopulmonary bypass (CPB) predisposes patients to cerebral hypoperfusion through systemic inflammation, ischemia–reperfusion injury, and blood–brain barrier disruption [4–6]. Intraoperative fluctuations in hemodynamics further destabilize cerebrovascular tone, and impaired autoregulation—reported in 10%–30% of CPB patients—is linked to stroke, delirium, and mortality [7–9]. Pre-existing cardiovascular disease may exacerbate these risks [10, 11].

Nicardipine, a dihydropyridine calcium channel blocker, selectively dilates cerebral resistance vessels via L-type calcium channel inhibition [12]. It has been used to reverse refractory vasospasm [13], and systemic infusion has been associated with reduced delirium in critically ill patients [14].

However, its potential benefits during CPB remain largely unexplored. The unique CPB environment, characterized by hemodilution, nonpulsatile flow, and impaired autoregulation, poses major threats to cerebral perfusion. Whether low-dose nicardipine can improve cerebral blood flow without compromising systemic hemodynamics is unknown. This study was designed to investigate this question.

## Patients and methods

### Ethical approval

The study was approved by the Medical Ethics Committee of Nanjing First Hospital (KY20220805-03) and was registered with the Chinese Clinical Trial Registry (ChiCTR2200064688) on October 14, 2022, prior to patient enrollment. The protocol conformed to the principles of the Declaration of Helsinki, and all participants provided written informed consent. This manuscript also complies with the relevant CONSORT guidelines.

### Participants

Between April and July 2022, a total of 64 patients undergoing CABG with CPB at Nanjing First Hospital were enrolled in this study, with patient recruitment conducted during the same period and follow-up extending to 7 days postoperatively.

Participants were randomly assigned in a double-blind fashion into two equal groups using a digital table method: the nicardipine group (Group N, *n* = 32) and the control group (Group C, *n* = 32). Upon initiation of CPB, patients in Group N received a continuous intravenous infusion of nicardipine starting at 0.2 µg·kg⁻¹·min⁻¹. The infusion was titrated by 0.05–0.1 µg·kg⁻¹·min⁻¹ increments to maintain a mean arterial pressure (MAP) between 65 and 80 mmHg during CPB, while ensuring adequate carotid flow and stable regional cerebral oxygen saturation (rScO₂). The infusion rate was reduced or discontinued if MAP dropped below 60 mmHg or if excessive vasodilation occurred. The final maintenance dose for individual patients ranged between 0.2 and 0.5 µg·kg⁻¹·min⁻¹, with no patients exceeding this range. Patients in Group C received an equivalent volume of saline, and all infusions were discontinued at the end of CPB.

The exclusion criteria were as follows: (1) a history of cardiac surgery; (2) a history of cerebral hemorrhage or neurosurgical intervention; (3) moderate to severe carotid artery stenosis; (4) hepatic or renal dysfunction; (5) communication difficulties; and (6) substance abuse. In addition, certain patients were excluded from the statistical analysis due to the following reasons: (1) intraoperative circulatory instability; (2) the need for secondary CPB during surgery; (3) intraoperative or postoperative (within 7 days) death; and (4) voluntary withdrawal.

### Anesthesia methods

Upon entering the operating room, each patient had a peripheral venous line established and received oxygen via facemask at 5 L/min. The induction protocol included intravenous midazolam (0.05 mg/kg), sufentanil (0.8 µg/kg), cisatracurium (0.2 mg/kg), and propofol (1.0–1.5 mg/kg). Once adequate muscle relaxation was confirmed, endotracheal intubation was performed, and ventilation was managed using intermittent positive pressure ventilation. Ventilator settings were adjusted to deliver 70% inspired oxygen, a tidal volume of 6–8 mL/kg, a respiratory rate of 10–12 breaths per minute, and an inspiratory-to-expiratory ratio of 1:2, while maintaining an end-tidal CO₂ of 35–45 mmHg.

**Fig. 1:**
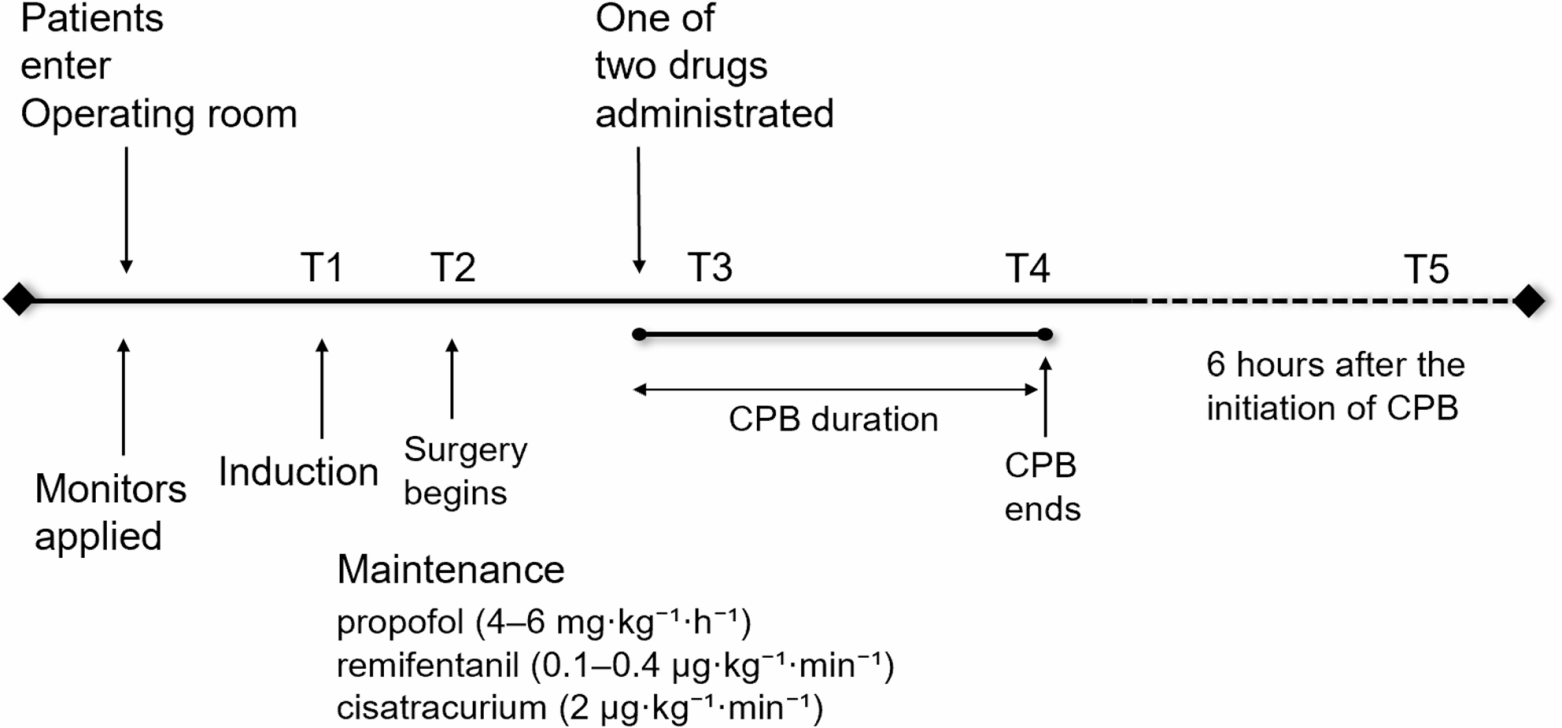
Schematic diagram of the study protocol

Throughout surgery, anesthesia was maintained with continuous infusions of propofol (4–6 mg·kg⁻¹·h⁻¹), remifentanil (0.1–0.4 µg·kg⁻¹·min⁻¹), and cisatracurium (2 µg·kg⁻¹·min⁻¹). After the initiation of CPB, the perfusion flow rate was maintained at 2.2–2.4 L·min⁻¹·m⁻², with a mean arterial pressure (MAP) of 65–80 mmHg, a nasopharyngeal temperature of 32–34 °C, hematocrit levels above 25%, and urine output exceeding 1.0 mL·kg⁻¹·h⁻¹. Intermittent sufentanil boluses were administered for analgesia, while the propofol infusion rate was titrated to maintain a bispectral index (BIS) of 40–50. Drug pumps for the Group N and C were prepared by unblinded anesthesia personnel using identical devices. These personnel pre-labeled each pump with a serial number corresponding to the group assignment determined by a computer-generated randomization table. A log documenting serial numbers, random numbers, and group assignments was prepared in duplicate and maintained independently by both the trial designer and the anesthesia personnel. Importantly, neither the trial designer nor those configuring the drug pumps participated in the trial administration. The attending anesthesiologist was responsible for overall anesthesia management, while a senior attending physician performed carotid ultrasound and transesophageal echocardiography monitoring. Data collection was handled by dedicated personnel who recorded relevant parameters and biochemical tests, and trained anesthesia staff conducted preoperative and postoperative MMSE. All participants remained blinded to their group assignments until unblinding upon trial completion.

### Anesthetic monitoring

Routine monitoring included electrocardiography oxygen saturation (SpO₂), and the BIS. A radial arterial canulation for invasive blood pressure monitoring and arterial blood gas analysis. Following endotracheal intubation, an esophageal temperature probe was inserted to monitor core temperature. a dual-lumen central venous catheter was placed via the right internal jugular vein for continuous central venous pressure (CVP) monitoring.

transesophageal echocardiography (TEE) was performed in all patients. In the mid-esophageal long-axis view of the aortic valve during systole, the diameter of the left ventricular outflow tract (D-LVOT) was measured. In the deep gastric five-chamber view, the sampling volume was positioned at the center of the LVOT near the aortic valve annulus, ensuring the ultrasound beam’s angle with blood flow was less than 15°. Velocity time integral (VTI) of the LVOT blood flow was obtained using pulse Doppler. Cardiac output (CO) was calculated with the formula: CO = HR × (VTI × πD²/4).

All TEE measurements were performed by the same qualified anesthesiologist, with each parameter recorded over three consecutive cardiac cycles and averaged. During CPB, the perfusion flow rate served as a surrogate for CO.

**Fig. 2:**
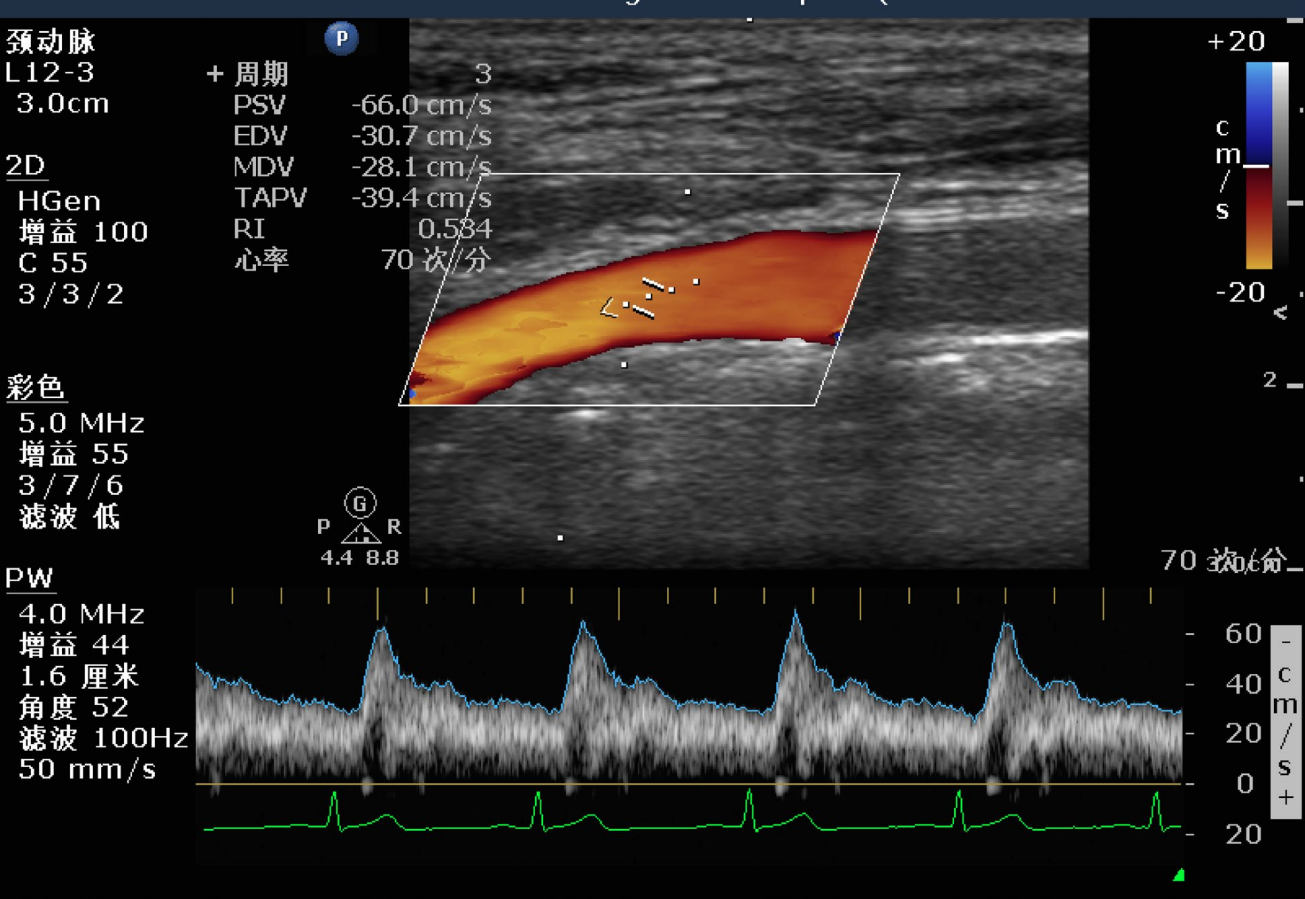
Ultrasound Image of PSV-ICA and EDV-ICA. PSV-ICA=Peak systolic velocity of the internal carotid artery; EDV-ICA= End-diastolic velocity of the internal carotid artery

**Fig. 3:**
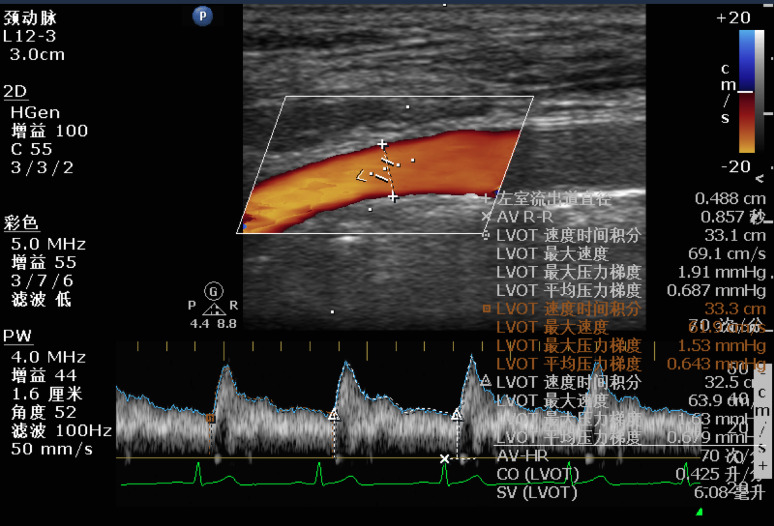
Ultrasound Image of ICA-VTI. ICA= Internal Carotid Artery; VTI= Velocity Time Integral

Upon entering the operating room, the patient’s head was slightly tilted to the right at a 30° angle, and an ultrasound probe was placed in the left supraclavicular fossa. In the short-axis view, the left common carotid artery (CCA) was identified, and the probe was then moved cephalad to the bifurcation to locate the left internal carotid artery (ICA). A measurement point was marked 1 cm above the bifurcation, and all subsequent measurements were taken at this site. Switching the probe to the longitudinal axis, the sampling volume was positioned at the center of the ICA while adjusting the ultrasound beam to form an angle of less than 60° with the blood flow. Once the blood flow velocity stabilized over 10–20 cardiac cycles, the image was frozen, and the peak systolic velocity (PSV-ICA) and end-diastolic velocity (EDV-ICA) were recorded. These measurements were repeated at least three times, and the average value was used for analysis (Figures 2 and 3).

The forehead was cleansed with an alcohol swab before an electrode for cerebral oxygen saturation (rScO_2_) was affixed for measurement. A cotton pad was placed over the electrode to minimize light interference. Baseline values were recorded before anesthesia induction.

This comprehensive monitoring protocol ensured a continuous and integrated assessment of the patient’s hemodynamic and oxygenation status throughout the surgical procedure.

### Study outcome

The primary outcomes of this study were PSV-ICA and EDV-ICA of the internal carotid artery, measured at four intraoperative time points (T1: before anesthesia induction; T2: at the start of surgery; T3: during CPB; T4: after CPB) (Fig. 1**).**

PSV-ICA reflects the forward systolic driving force of cerebral blood flow, while EDV-ICA represents diastolic perfusion and vascular resistance. Together, these parameters provide a comprehensive evaluation of carotid blood flow and cerebral perfusion, and are widely used as sensitive, noninvasive indicators of intraoperative cerebral hemodynamics.

Secondary outcomes included: (a) Plasma NSE concentrations (T1 and 6 h post-CPB, T5) as a biochemical marker of neuronal injury. (b) MMSE scores preoperatively and 7 days postoperatively as a measure of cognitive function. (c) rScO₂ at T1–T4, reflecting global cerebral oxygen balance. (d) Conventional hemodynamic parameters (HR, MAP, CVP, CO), arterial blood gases, and perioperative outcomes (vasopressor use, ventilation duration, ICU stay, hospitalization length).

Safety outcomes included hypotension (MAP < 60 mmHg lasting ≥ 1 min or requiring vasopressor treatment), arrhythmias (detected by intraoperative ECG), and major bleeding (defined as re-exploration). All adverse events were prospectively monitored intraoperatively.

### Statistical analysis

Sample size estimation was based on preliminary data for PSV-ICA at T4 (62.8 ± 16.7 cm/s in Group N vs. 51.5 ± 13.8 cm/s in Group C). With α = 0.05 and power (1–β) = 0.80, the required sample size was 30 subjects per group. Allowing for a 10% dropout rate, 33 patients per group were recruited.

In practice, one patient underwent off-pump CABG and one patient required a second CPB, leaving 32 patients per group for the final analysis.

We analyzed PSV and EDV across four peri-CPB time points (T1–T4) using linear mixed-effects models (LMMs) with fixed effects for group, time, and their interaction, and random intercepts for patients to account for within-subject correlation. Estimated marginal means (EMMs) were obtained using the emmeans package in R. Pairwise group comparisons at each time point were performed based on model-derived EMMs. To control for multiplicity across repeated contrasts, Bonferroni correction was applied, and adjusted P values < 0.05 were considered statistically significant. All other outcomes were analyzed as secondary or exploratory with a significance threshold of *P* < 0.05.

For secondary outcomes (MMSE and NSE), analysis of covariance (ANCOVA) was performed with the baseline value as a covariate. To account for multiple testing, p values were further adjusted using both the Benjamini–Hochberg false discovery rate (FDR) procedure and the Bonferroni correction. Both raw and adjusted p values are reported.

Intraoperative hemodynamic parameters (HR, MAP, CVP, CO, and other variables) were summarized descriptively by group and time point, presented as mean ± standard deviation or median (interquartile range) as appropriate. No formal hypothesis testing was performed for these variables, as they were not prespecified outcomes, but they provide contextual information regarding perioperative stability.

Normally distributed variables were presented as mean ± standard deviation and compared between groups using independent-samples t tests. Non-normally distributed variables were expressed as median (interquartile range). All statistical analyses were conducted using R version 4.3.1 (R Foundation for Statistical Computing, Vienna, Austria) and SPSS software (SPSS, Chicago, IL).

## Results

Of the 70 patients screened for eligibility, 64 patients met the inclusion criteria and were randomly allocated into the control group (*n* = 32) or the nicardipine group (*n* = 32). All patients received the allocated interventions and were included in the analyses (Fig. 1). The two groups were generally comparable regarding the baseline characteristics (Table 1). No significant differences were observed in gender distribution (male: 65.6% vs. 62.5%, *P* = 0.794), age at operation (68.7 ± 5.6 vs. 69.5 ± 5.2 years, *P* = 0.535), or preoperative ejection fraction (58.8 ± 3.7% vs. 59.5 ± 3.4%, *P* = 0.404). The distributions of ASA and NYHA classifications were also similar between groups. Intraoperative and perioperative variables, including CPB duration (105.3 ± 18.9 vs. 110.2 ± 16.3 min, *P* = 0.274), aortic cross-clamp time (78.3 ± 15.8 vs. 73.6 ± 16.4 min, *P* = 0.245), and total surgery time (266.5 ± 33.7 vs. 256.2 ± 43.3 min, *P* = 0.291), did not differ significantly.

**Table 1:** Demographic data and baseline characteristics

Characteristics	Group N (*n* = 32)	Group C (*n* = 32)	*P*-value
Gender (%)			
Male	21 (65.6)	20(62.5)	0.794
Female	11 (34.4)	12 (37.5)	
Age at operation (yr)	68.7 ± 5.6	69.5 ± 5.2	0.535
BMI (kg/m²)	23.5 ± 2.6	23.3 ± 2.3	0.706
Preoperative EF	58.8 ± 3.7	59.5 ± 3.4	0.404
ASA grade (%)			
III	4 (12.5)	2 (6.3)	0.668
IV	28 (87.5)	30 (93.8)	
NYHA grade (%)			
III	14 (43.8)	13 (40.6)	0.800
IV	18 (56.3)	19 (59.4)	
CPB duration (min)	105.3 ± 18.9	110.2 ± 16.3	0.274
Aortic cross-clamp time (min)	78.3 ± 15.8	73.6 ± 16.4	0.245
Surgery time (min)	266.5 ± 33.7	256.2 ± 43.3	0.291
Dose of epinephrine (mg)	0.9 ± 0.2	0.8 ± 0.2	0.290
Dose of phenylephrine (µg)	900 200	850 250	0.287
ETT duration (hour)	12.9 ± 2.6	13.0 ± 2.7	0.889
ICU stay (hour)	39.1 ± 8.9	41.5 ± 9.8	0.310
Hospital stays (day)	10.7 ± 1.2	10.2 ± 1.3	0.134

BMI = body mass index; EF = ejection fraction; ASA = American Society of Anesthesiologists; NYHA = New York Heart Association; CPB = cardiopulmonary bypass; ETT = endotracheal intubation; ICU = intensive care unit

**Fig. 4:**
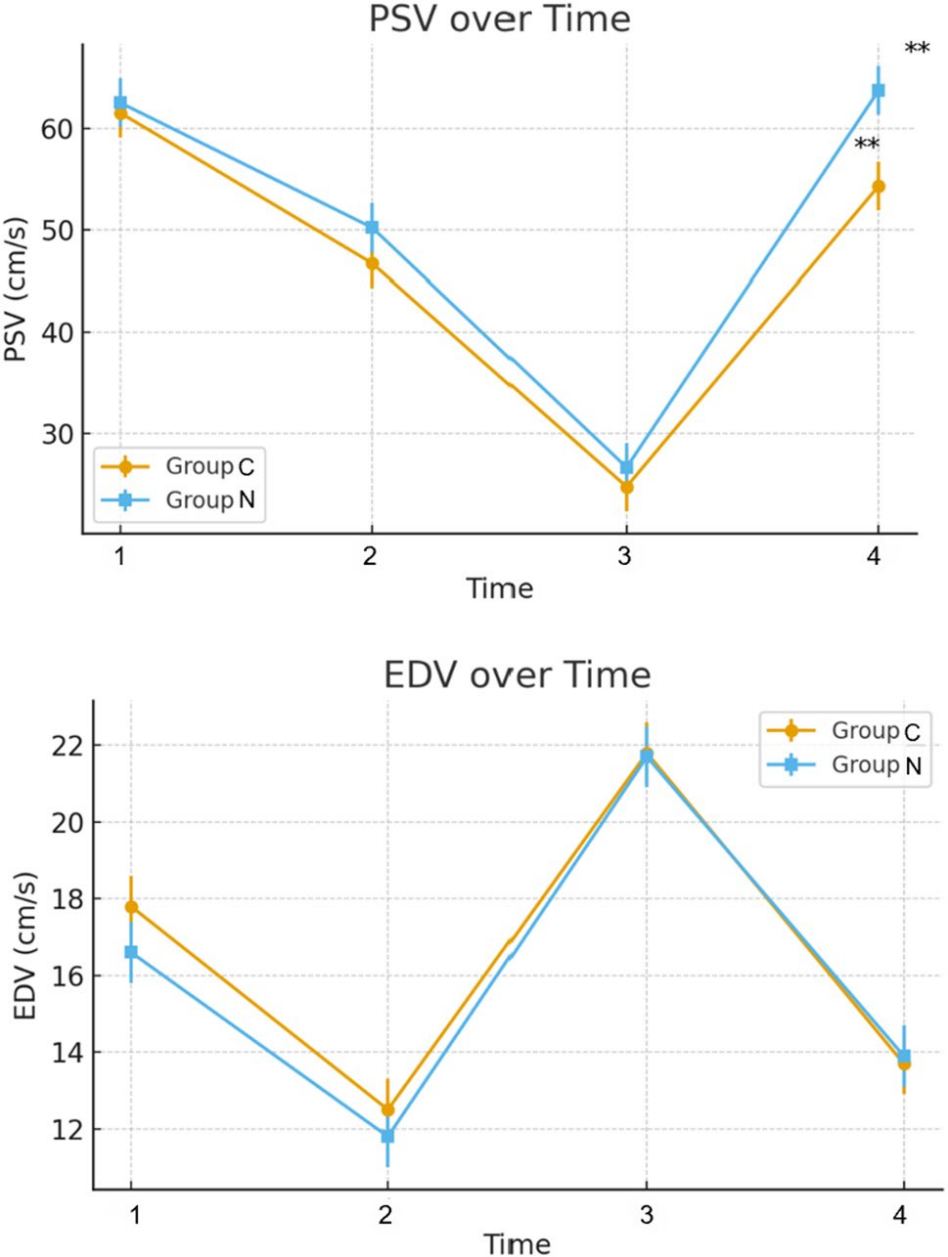
Line plots of PSV and EDV at different time points in Group N and Group C. PSV= peak systolic velocity; EDV= end-diastolic velocity. **indicates a statistically significant difference between the two groups at the corresponding time point after Bonferroni correction (adjusted P < 0.025). Data are presented as mean ± SE

Both groups experienced a marked decline in PSV-ICA following initiation of surgery, with values decreasing from T1 to T2 and reaching the lowest point at T3. By the end of CPB (T4), PSV-ICA rebounded toward baseline in both cohorts (Fig. 4); however, the nicardipine group showed a significantly greater recovery than controls (63.7 ± 15.2 vs. 54.3 ± 13.9 cm/s; Bonferroni *P* = 0.025) (Table 2).

**Table 2:** Comparison of carotid artery blood flow parameters at different time points between two groups

Time point	Group	PSV (cm/s)	Contrast (95% CI)	*P* value	Bonferroni *P*-value	EDV (cm/s)	Contrast (95% CI)	*P* value	Bonferroni *P*-value
T1	N	62.6 ± 17.4	–1.0 (–7.7, 5.7)	0.770	> 0.05	16.6 ± 3.8	1.2 (–1.0, 3.4)	0.286	> 0.05
	C	61.6 ± 14.4				17.8 ± 5.2			
T2	N	50.2 ± 15.4	–3.5 (–10.2, 3.2)	0.303	> 0.05	11.8 ± 2.3	0.8 (–1.4, 3.0)	0.480	> 0.05
	C	46.7 ± 14.4				12.6 ± 2.9			
T3	N	26.7 ± 7.0	–1.9 (–8.6, 4.9)	0.586	> 0.05	21.7 ± 6.2	0.1 (–2.1, 2.3)	0.954	> 0.05
	C	24.8 ± 7.5				21.8 ± 7.1			
T4	N	63.7 ± 15.2	–9.4 (–16.2, − 2.7)	0.006	0.025*	13.9 ± 2.6	–0.3 (–2.5, 1.9)	0.812	> 0.05
	C	54.3 ± 13.9				13.7 ± 3.4			

PSV = peak systolic velocity of left internal carotid artery; EDV = end-diastolic velocity of left internal carotid artery

Values are estimated marginal means (EMMs) ± standard error derived from linear mixed-effects models. Pairwise group comparisons were obtained from model-derived EMMs, with Bonferroni correction applied for multiple time points. A significant between-group difference in PSV was observed at T4 (adjusted *P* = 0.025), whereas no differences were detected for EDV at any time point

EDV-ICA exhibited a similar peri-CPB pattern, with significant reductions during bypass (T1–T2) and partial recovery at T4 (Fig. 4). In the nicardipine group, EDV-ICA at T4 was significantly higher than its own T1 and T2 values, although between-group differences in EDV-ICA did not reach statistical significance after Bonferroni correction (Table 2).

During the peri-CPB period, hemodynamic and metabolic parameters were comparable between the two groups (Table 3). No significant differences were detected in heart rate, MAP, CVP, cardiac output, arterial PaO₂, hemoglobin, hematocrit, temperature, lactate, or glucose at any time point, and both groups showed similar trajectories across T1–T4.

**Table 3:** Comparison of basic monitoring parameters at different time points between two groups

Time point	T1	T2	T3	T4
HR (bpm)				
Group N	74.4 ± 13.4	56.9 ± 7.7		81.7 ± 11.1
Group C	75.9 ± 12.8	59.8 ± 8.4		81.3 ± 8.6
MAP (mmHg)				
Group N	91.3 ± 10.8	71.3 ± 6.5	69.7 ± 3.3	71.2 ± 6.5
Group C	96.5 ± 12.0	71.8 ± 6.6	70.5 ± 2.9	70.5 ± 6.3
CVP (mmHg)				
Group N		7.4 ± 1.9	−3.8 ± 1.2	9.6 ± 2.6
Group C		6.7 ± 1.8	−3.6 ± 1.3	8.6 ± 2.9
CO (L/min)				
Group N		3.4 ± 0.9	4.7 ± 0.3	5.8 ± 1.1
Group C		3.2 ± 0.9	4.7 ± 0.4	5.3 ± 1.5
PaO_2_ (mmHg)				
Group N	222.2 ± 48.5	254.4 ± 30.3	257.5 ± 32.0	249.4 ± 82.3
Group C	208.6 ± 41.8	247.3 ± 29.9	256.8 ± 25.4	248.0 ± 85.9
Hb (g/L)				
Group N	140.7 ± 20.9	126.4 ± 19.8	86.9 ± 10.3	96.8 ± 8.4
Group C	140.0 ± 17.0	125.2 ± 16.6	83.8 ± 8.9	101.0 ± 10.9
Hct (%)				
Group N	43.88 ± 6.41	40.59 ± 5.88	27.50 ± 1.52	28.13 ± 1.79
Group C	43.91 ± 5.65	40.84 ± 5.30	27.41 ± 1.46	28.16 ± 2.19
Temperature (°C)				
Group N		36.1 ± 0.3	32.9 ± 0.5^†^	35.7 ± 0.2
Group C		36.2 ± 0.2	32.9 ± 0.4^†^	35.7 ± 0.3
Lac (mmol/L)				
Group N	1.0 ± 0.3	1.1 ± 0.3	1.1 ± 0.3	1.4 ± 0.4
Group C	1.0 ± 0.4	1.0 ± 0.3	1.0 ± 0.3	1.3 ± 0.5
Glu (mmol/L)				
Group N	6.2 ± 0.9	6.5 ± 0.8	7.4 ± 1.1	7.8 ± 1.1
Group C	6.0 ± 0.8	6.3 ± 0.8	7.1 ± 1.0	7.4 ± 1.4
rScO_2_ (%)				
Group N	71.0 ± 4.8	65.5 ± 4.9	62.7 ± 3.9	70.2 ± 4.5
Group C	70.2 ± 5.3	65.8 ± 5.0	60.1 ± 3.8	66.7 ± 4.9

HR = heart rate; MAP = mean arterial pressure; CVP = central venous pressure; CO = cardiac output; PaO_2_ = partial pressure of oxygen; Hb = hemoglobin; Hct = hematocrit; Lac = lactic acid; Glu = glucose; rScO2 = regional cerebral saturation oxygenation

After adjustment for baseline values, no significant between-group differences were observed in MMSE (raw *p* = 0.398) or NSE (raw *p* = 0.509). These results remained nonsignificant after correction for multiple comparisons (FDR-adjusted q = 0.509 for both; Bonferroni-adjusted *p* = 0.795 for MMSE and *p* = 1.000 for NSE) (Table 4).

**Table 4:** Comparative analysis of NSE levels and MMSE scores at different time points in groups N and C

Time Point	T1	T5			
NSE (ng/mL)			**Raw p (ANCOVA)**	**FDR q**	**Bonferroni p**
Group N	10.2 ± 2.8	37.8 ± 3.8	0.398	0.509	0.795
Group C	10.9 ± 3.6	38.4 ± 4.0			
**Time point**	**Preoperative day 1**	**Postoperative day 7**			
Score of MMSE			**Raw p (ANCOVA)**	**FDR q**	**Bonferroni p**
Group N	28 (25, 31)	27 (25, 29)	0.509	0.509	1.000
Group C	28 (26, 30)	27.5 (25, 30)			

NSE = neuron-specific enolase, MMSE = Mini-Mental State Examination

Values represent raw p values from ANCOVA (adjusting for baseline). Multiple testing correction was performed using the Benjamini–Hochberg false discovery rate (FDR, reported as q values) and Bonferroni method (Bonferroni-adjusted p values). None of the secondary outcomes (MMSE, NSE) reached statistical significance after adjustment

No adverse events, including hypotension, arrhythmias, or major bleeding, were observed in either group during the perioperative period.

## Discussion

In this randomized controlled trial of patients undergoing CPB, we observed that continuous low-dose nicardipine infusion was associated with a tendency toward greater recovery of PSV-ICA at the end of CPB compared with controls. EDV-ICA followed a similar perioperative pattern, although between-group differences did not reach statistical significance after correction for multiple comparisons.

Cerebral blood flow (CBF) normally constitutes 10–15% of cardiac output, with approximately 75% delivered via the internal carotid arteries (ICAs) and 25% via the vertebral arteries. Autoregulatory mechanisms maintain stable CBF by adjusting arteriolar diameter in response to perfusion pressure changes, but this capacity has finite limits; once exceeded, cerebral perfusion falls and cognitive function may be compromised [15]. Aging further impairs perfusion chiefly through reductions in flow velocity rather than vessel caliber [16]. Duplex ultrasonography of the ICA provides a rapid, noninvasive measure of carotid flow (PSV-ICA and EDV-ICA), while near-infrared spectroscopy (NIRS) continuously monitors rScO₂, reflecting anterior and middle cerebral artery perfusion. Unilateral ICA clamping, for example, causes a 20% drop in rScO₂ and correlates with impaired verbal function, underscoring the tight link between carotid flow, NIRS readings, and neurocognitive outcomes [17, 18].

In our study, conventional hemodynamic (HR, MAP, CO) and laboratory (hemoglobin, PaCO₂) parameters remained comparable between groups at all time points, isolating the effect of nicardipine. During CPB, PSV-ICA declined in both groups; however, following separation from CPB, the nicardipine group exhibited a significantly greater recovery of PSV-ICA compared with controls (*P* < 0.025). These data suggest that low-dose nicardipine infusion during CPB augments carotid flow, in agreement with reports of intrathecal nicardipine improving cerebral perfusion and oxygenation in vasospasm patients [19].

Despite these improvements in cerebral perfusion, we did not observe significant differences in postoperative MMSE scores between groups. This discrepancy highlights the complex and multifactorial pathophysiology of POCD. Large-scale analyses indicate delirium rates of 18–24% and delayed neurocognitive recovery in 43% of CABG patients, with POCD persisting in 19–25% at 4–12 months post-surgery [20, 21]. Cognitive decline following CABG has been attributed to a wide range of mechanisms, including cerebral hypoperfusion, embolic events, systemic inflammation, and patient-related risk factors [22]. In both surgical and non-surgical populations, lower carotid flow velocities correlate with reduced MMSE scores [23, 24]. In our study, cognitive assessment was limited to MMSE at postoperative day 7, which may not capture delayed or persistent POCD. Therefore, the absence of detectable differences between groups should be interpreted cautiously, reflecting early postoperative trends rather than long-term neurocognitive outcomes. The multifactorial etiology of POCD and the short follow-up period likely contributed to this finding.

NSE, a glycolytic enzyme localized to the neuronal cytoplasm, is a highly specific biomarker of neuronal damage and is routinely used to detect subclinical brain injury following coronary and carotid revascularization [25]. Serum NSE levels typically rise within 6 h of neuronal insult, decline by 24 h, and peak again at 72 hours [26, 27]. In our cohort, both groups exhibited a significant elevation in NSE 6 h after the initiation of CPB compared with baseline (pre-induction), confirming that CPB per se induces a degree of cerebral injury. This finding aligns with Barbu et al., who reported a transient postoperative increase in NSE, peaking 2 h after surgery, even in the absence of overt neurological symptoms [28]. The absence of a statistically significant difference between the nicardipine and control arms may reflect the low dose and timing of nicardipine infusion, as well as the limited postoperative monitoring window for NSE.

In our study, NSE was measured at 6 h after CPB. This early time point was selected based on prior evidence that NSE rises rapidly within hours of neuronal injury and provides a sensitive marker for acute brain injury detection. However, NSE is known to exhibit a biphasic pattern, with a secondary peak often occurring at 48–72 h postoperatively. The lack of additional later measurements represents an important limitation of our study, as it may have led to underestimation of delayed or secondary neuronal injury. Future investigations should incorporate serial sampling at multiple postoperative time points (6 h, 24 h, 48–72 h) to better characterize the temporal profile of neuronal damage in this setting.

This trial integrated multimodal assessments—including biochemical markers, duplex ultrasonography, NIRS, and neurocognitive testing—to provide a comprehensive evaluation of cerebral protection during CPB. Limitations include its single-center design, relatively small sample size, unilateral ICA monitoring without vertebral assessment, and short-term postoperative cognitive evaluation. Future studies should investigate alternative dosing strategies, prolonged infusions, and extended cognitive follow-up to clarify the neuroprotective potential of nicardipine.

In summary, low-dose nicardipine infusion during CPB augmented cerebral blood flow velocity and regional oxygenation, suggesting potential neuroprotective benefits. However, these improvements did not translate into measurable short-term cognitive advantages, and the short follow-up limits inference regarding long-term neurocognitive outcomes. Further studies with larger cohorts and longer follow-up are warranted to determine whether nicardipine can improve long-term neurocognitive outcomes after CABG.

## Data Availability

No datasets were generated or analysed during the current study.
